# The Cracking of Teeth in Soldering

**Published:** 1894-05

**Authors:** L. P. Haskell

**Affiliations:** CHICAGO.


ARTICLE VII.

### THE CRACKING OF TEETH IN SOLDERING.

BY L. P. HASKELL, D. D. S., CHICAGO.

In the last number of the Review, in an article on "Setting Bridges;" the remark is made by Dr. McCandless that those who say they do not crack teeth in soldering "do not know whether the teeth are cracked or not," that "they are often cracked and do not show." I have been soldering teeth for nearly fifty years; think I "know a good thing when I see it," and especially whether a tooth is cracked or not. A crack can *always* be seen when it is *dry*, but often while moisture is in, it does not appear.

He asserts that it is impossible to solder teeth in crown and bridge work without cracking more or less teeth. I reply that there is no need of teeth cracking in soldering, and furthermore that no special care is needed to prevent it.

Many of the students at our school, coming from the dental colleges, tell me that there they have much trouble from this cause and wish to know how to prevent it. They, however, go on with their work soldering partial sets of gum teeth, crowns and bridges; some of them having no cracks and others but few, and this too in the early use of the blowpipe.

In my own experience, and taking no pains to avoid it, I do not remember the time when I ever had a tooth crack.

Perhaps a few suggestions may not be amiss. If dentists would consider that "cross" pin teeth are more apt to crack than the perpendicular or "straight" pin teeth, they would use fewer of them. They are not only more liable to crack in soldering, but are not as strong for wear. This fact is so apparent it has been a great wonder to me that at the dental depots, when inquiring for "straight" pins and wondering there were so few, I am told that "the dentists all ask for 'cross pins.'" Use less of them, gentlemen, and you will have fewer cracks in soldering and less breakage in wear.

Do not rivet your pins, as the solder flows only upon the head of the pin; split the pin, and if the hole is larger than the pin, so much the better, for the solder flows down inside and holds the stronger.

Never heat up the case until the borax and solder have been applied. Heat up over the large burner, slowly at first, and then turning on the full head; take about one-half hour. The heat should (in case of a plate) be thrown by the blow-pipe upon the plate first, so that it may be as hot as the backings, as they will heat up as a matter of course from their exposed position. Then throw the heat directly upon the solder. If necessary to apply more solder do so, but not more borax, as there is danger of cracking the teeth.

Many dentists experience much difficulty in soldering by using the miserable little jewelers' blowpipes, which are not at all suitable for dentists' use, there not being a sufficient volume of flame, and the mouth aperture being so small that it has to be taken inside the lips, thereby causing the muscles to become tired. To remedy this I induced the dental goods manufacturers to make a larger blowpipe —the mouth-piece to be pressed against the lips—which makes soldering much easier.

Another difficulty is found in not having a suitable soldering burner. Formerly I wound fine binding wire over the end of the gas pipe so as to break the force of the gas jet. But I am now using a burner made for the purpose, which some have called the "Haskell burner." Many use the automatic blowpipe, but I have found that beginners succeed better with the blowpipe, and for my own use far prefer it.—*Dental Review.*

